# Establishment of an ultrasound-guided rabbit model of hepatic inflammatory pseudotumor and contrast-enhanced ultrasound pattern analysis

**DOI:** 10.3389/fvets.2026.1904243

**Published:** 2026-09-30

**Authors:** Zhi-long Liu, Qian Zhang, Qin Lu, Yan-jing Zhang, Li Li, Yu-fang Zhao, Guo-lin Yin, Xiao-hui Yan, Yan-hua Zhang, Li-ping Liu

**Affiliations:** 1 Department of Ultrasound, The Fifth Clinical Medical College of Shanxi Medical University, Taiyuan, Shanxi, China; 2 Department of Ultrasound Intervention, First Hospital of Shanxi Medical University, Taiyuan, Shanxi, China; 3 Department of Neurology, First Hospital of Shanxi Medical University, Taiyuan, Shanxi, China; 4 Department of Pathology, The Fifth Clinical Medical College of Shanxi Medical University, Taiyuan, Shanxi, China

**Keywords:** contrast-enhanced ultrasound (CEUS), inflammatory pseudotumor, liver, model, rabbit

## Abstract

**Objective:**

The aim of this study was to develop a rabbit model of inflammatory pseudotumor of the liver (IPTL) using an ultrasound-guided implantation technique and to assess its feasibility, safety, and imaging characteristics in comparison with a surgical embedding approach.

**Methods:**

Twenty-six New Zealand rabbits were randomly assigned to one of three groups: the ultrasound-guided implantation group (Experimental Group), the surgical embedding group (Surgical Group), or a blank control group. IPTL lesions were induced using a combination of gelatin sponge and Freund’s complete adjuvant. Parameters assessed included operative duration, lesion modeling success rate, findings from conventional ultrasound and contrast-enhanced ultrasound (CEUS), laboratory indicators, and histopathological outcomes.

**Results:**

All animals survived without severe complications. The lesion modeling success rates were 90% in the Experimental Group and 95% in the Surgical Group (*p* > 0.05), while operative duration was significantly reduced in the Experimental Group (*p* < 0.05). On conventional ultrasound, IPTL lesions in both experimental groups presented as hyperechoic nodules with well-defined margins. CEUS indicated three distinct enhancement patterns: isoenhancement, hypoenhancement, and rapid washout. Laboratory testing demonstrated no significant differences in serum hyaluronic acid or liver function markers between the Experimental and control groups; however, the Surgical Group exhibited significantly elevated hyaluronic acid levels (*p* < 0.05). Immunohistochemical analysis demonstrated lower microvessel density in hypoenhanced nodules compared to those with other enhancement patterns.

**Conclusion:**

The ultrasound-guided implantation technique is a safe, efficient, and reproducible method for modeling IPTL in rabbits. The resulting lesions exhibit histopathological and CEUS characteristics that closely resemble those observed in human IPTL, supporting its utility as an experimental platform for further research into the pathophysiology and imaging features of IPTL.

## 1 Introduction

Inflammatory pseudotumor of the liver (IPTL) is a rare, non-specific inflammatory hepatic mass characterized by dense infiltration of inflammatory cells, including lymphocytes and plasma cells, accompanied by stromal alterations (1). IPTL is infrequently encountered in clinical practice and is not associated with distinct epidemiological or clinical features (2, 3). Current understanding of IPTL remains limited, as most of the available literature consists of isolated case reports (4). Imaging findings of IPTL are heterogeneous. On CEUS, lesions often exhibit portal venous phase washout, a pattern that can be misinterpreted as malignancy, particularly intrahepatic cholangiocarcinoma (ICC) or hepatocellular carcinoma (HCC), frequently resulting in unnecessary surgical procedures (5–8). As a result, IPTL continues to represent a significant diagnostic challenge in clinical hepatology.

Ultrasound, particularly CEUS, is the preferred first-line imaging modality for the evaluation of liver diseases. In recent years, both conventional ultrasound and CEUS have been increasingly employed for the detection and characterization of hepatic lesions (9, 10). However, the differentiation of IPTL from other focal hepatic lesions remains challenging, and the diagnostic performance of ultrasound in the context of IPTL has yet to be fully clarified (11). Also, the pathophysiological mechanisms underlying IPTL are poorly understood, and standardized diagnostic criteria have not been established (12). Therefore, the development of a reliable and reproducible animal model is essential for advancing the understanding of IPTL pathophysiology, characterizing its imaging features, and facilitating the investigation of novel diagnostic and therapeutic approaches.

To date, no widely accepted animal model for IPTL has been established (13). Although previous studies have developed rabbit models that replicate certain histopathological features of IPTL, these models were mainly established using surgical implantation methods and showed relatively uniform CEUS enhancement patterns, typically isoenhancement relative to surrounding hepatic parenchyma, limiting their ability to reflect the diverse enhancement characteristics observed in human cases. In the present study, we developed a modified ultrasound-guided implantation approach based on previously established IPTL modeling principles. This technique enables real-time visualization of the implantation process, accurate deposition of modeling materials, and reduced procedural trauma (14, 15). Furthermore, unlike previous models that mainly demonstrated relatively homogeneous CEUS enhancement patterns, our model reproduced heterogeneous CEUS manifestations, providing a potential platform for investigating imaging-pathological correlations in IPTL.

## 2 Materials and methods

### 2.1 Experimental animals

A total of 26 healthy New Zealand white rabbits of the same age (mean body weight: 2.5 ± 0.5 kg; mixed sex) were procured from Qingdao Kangda Animal Experimental Center (Qingdao, China). All animals underwent a 7-day acclimatization period under controlled environmental conditions, including regulated temperature, humidity, and fixed light–dark cycles, with unrestricted access to food and water. Following acclimatization, the rabbits were randomly allocated into the experimental group and surgical control group using a simple randomization method by drawing lots before the modeling procedure: the ultrasound-guided implantation group (Experimental Group, *n* = 10), the surgical embedding group (Surgical Group, *n* = 10), and the blank control group (*n* = 6).

All procedures involving animals were carried out in strict accordance with the guidelines for the care and use of laboratory animals. Approval of the study protocol was granted by the Ethics Committee of Shanxi Medical University (Approval No. KYLL-2023-132).

### 2.2 Modeling

#### 2.2.1 Ultrasound-guided implantation

Two inflammatory pseudotumor nodules were induced in each rabbit using the following procedure: ① Anesthesia was induced via intramuscular injection of Shutai 50 (2 mg/kg, 0.04 mL/kg) and Su-Mian-Xin II (6 mg/kg, 0.06 mL/kg) into the hindlimb musculature (16). ② Following anesthesia induction, each rabbit was placed in the supine position on the operating table with the upper abdomen exposed. Thoracoabdominal hair was shaved, the skin was disinfected, and a sterile drape was applied. ③ A gelatin sponge was cut into approximately 1mm^3^ cubes. Using ophthalmic forceps, 5 of these fragments were loaded into the distal end of an 18G puncture sheath. A 1-mL syringe was then connected to the sheath, and the tip was immersed in Freund’s complete adjuvant (FCA; Sigma, USA). FCA was drawn into the sheath until it was fully filled (Figure 1A). An additional 0.3 mL of FCA was prepared in a separate syringe for subsequent injection. ④ The puncture route was selected under ultrasound guidance to avoid visible intrahepatic vessels, and the needle tip position was confirmed before implantation. Once the sheath was secured, the stylet was advanced to deliver the gelatin sponge–FCA mixture into the hepatic parenchyma (Figure 1B). The stylet was then withdrawn, and without repositioning the sheath, 0.3 mL of FCA was slowly injected into the same site to allow full infiltration of the gelatin sponge and dispersion into the adjacent hepatic tissue (Figure 1C). The needle was removed, and gentle compression was applied to the puncture site for 2–3 min. The procedure duration was recorded, defined as the time interval from skin preparation after anesthesia to completion of needle withdrawal and compression hemostasis. ⑤ Postoperatively, intramuscular injections of penicillin (4 × 10^4^ U) were administered daily for three consecutive days to prevent infection.

**Figure 1:**
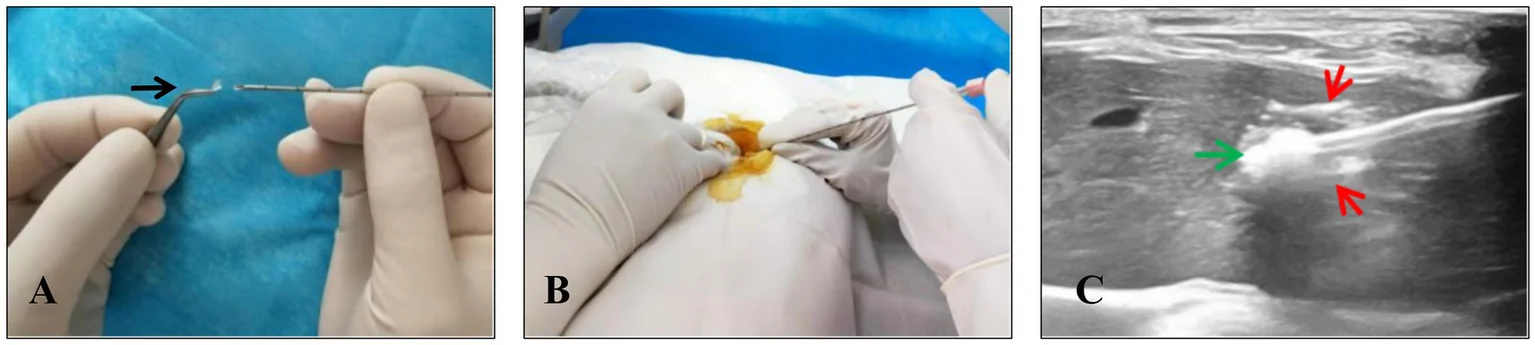
Ultrasound-guided implantation modeling. **(A)** Gelatin sponge cubes (1 mm^3^) were loaded into the tip of an 18G puncture sheath using ophthalmic forceps. **(B)** Under ultrasound guidance, the sheath was positioned at the target site in the liver, and the stylet was advanced to implant the gelatin sponge along with Freund’s complete adjuvant (FCA) into the hepatic tissue. **(C)** After delivering the gelatin sponge particles (green →) with the stylet, a 1-mL syringe was attached to the needle hub, and a small volume of FCA was slowly injected, allowing it to permeate the gelatin sponge and diffuse gradually into the surrounding liver parenchyma (red →).

#### 2.2.2 Surgical embedding

1. Anesthesia and preoperative preparation: Anesthesia was induced using the same protocol as previously described. Following induction, each rabbit was positioned in the supine position. The upper abdomen was shaved and disinfected. A sterile surgical drape was applied to maintain aseptic conditions.
2. Surgical procedure: A midline abdominal incision approximately 4 cm in length was made along the linea alba. The abdominal wall was incised layer by layer to expose the liver, and the left and right median lobes were gently mobilized. At the thickest area of each lobe, a short tunnel was created using a hemostat. After achieving local hemostasis through gentle compression, 5 pieces of gelatin sponge (1 mm^3^) were inserted into the tunnel, followed by injection of 0.3 mL of FCA into the same site. The needle was withdrawn, and gentle compression was applied for 2–3 min to achieve hemostasis.
3. Wound closure: Upon completion of the implantation, the hepatic lobes were repositioned, and the abdominal incision was closed in layers with absorbable sutures.
4. Postoperative care: Intramuscular injections of penicillin were administered at the same dosage as in the experimental group.

### 2.3 Evaluation

#### 2.3.1 Ultrasound assessment

Based on preliminary data, imaging assessments were conducted 3 weeks following the modeling procedure (17). Successful IPTL modeling was defined as a distinct focal hepatic nodule visible on ultrasound. All examinations were carried out using a Resona 9 ultrasound system (Mindray, Shenzhen, China). Conventional ultrasound was performed with an L15-3WU linear array transducer, while CEUS examinations were conducted using an L9-3U linear array transducer. Two independent sonographers with more than 10 years of clinical experience independently reviewed all ultrasound images while blinded to the pathological findings. Any disagreements in image interpretation were resolved by consensus with the assistance of a third experienced sonographer.

For B-mode ultrasound, the maximum diameter of the lesion, shape, echogenicity, margin definition, and color Doppler flow imaging (CDFI) characteristics were recorded. Blood flow was classified using Adler’s grading system, a 0–3 semi-quantitative visual scale was adopted to evaluate lesion vascularity on color Doppler images. Grade 0 denotes absent blood flow, Grade 1 minimal punctate flow pixels, Grade 2 moderate visible small vessels, and Grade 3 marked hypervascularity with ≥4 distinct vessels (18).

For CEUS examinations, a contrast-specific imaging mode with a low mechanical index (MI = 0.086) was used to minimize microbubble disruption. SonoVue™ (Bracco SpA, Milan, Italy) was administered intravenously via the marginal ear vein at a dose of 0.1 mL/kg, followed by a 1 mL saline flush. The following quantitative CEUS parameters were measured: peak intensity (PI), defined as the maximum enhancement intensity during the observation period; arrival time (AT), defined as the time from contrast agent injection to the initial enhancement of the lesion; rise time (RT), defined as the time interval from the beginning of enhancement to peak enhancement; time to peak (TTP), defined as the time from contrast agent injection to peak enhancement; and washout time (WT), defined as the time interval from peak enhancement to the onset of lesion washout. The degree of enhancement was measured with reference to the liver parenchyma surrounding the lesion. Washout was defined as a visually assessed temporal reduction in lesion enhancement relative to the surrounding liver parenchyma (19).

#### 2.3.2 Laboratory testing

Following CEUS, 4 mL of blood was collected from the heart under ultrasound guidance. The samples were placed in serum separator tubes and centrifuged at 3000 rpm to isolate serum. The following biochemical indicators were measured: hyaluronic acid (HA), alanine aminotransferase (ALT), aspartate aminotransferase (AST), and total bilirubin (TBIL).

#### 2.3.3 Histopathological examination

At the conclusion of the experiment, animals were euthanized under anesthesia via marginal ear vein injection of overdose sodium pentobarbital (≥100 mg/kg). Lesions were sectioned along the maximum diameter to include both lesion and adjacent liver parenchyma, fixed in 10% neutral-buffered formalin, embedded in paraffin, and sectioned. The sections were stained with hematoxylin and eosin (H&E), sealed with neutral resin, and examined by light microscopy.

For immunohistochemical (IHC) analysis, a rabbit polyclonal anti-CD34 antibody was used as the primary antibody, and a universal anti-rabbit/mouse antibody served as the secondary antibody. All reagents were obtained from Proteintech (Wuhan, China), and staining procedures were carried out in accordance with the manufacturer’s instructions. Microvessel density quantification was performed in accordance with the internationally accepted Weidner counting method. All immunohistochemical slides were renumbered randomly and evaluated by two independent pathologists under blinded conditions. “Hotspot” regions were initially identified under low magnification (×10). Microvessel counts were subsequently performed under high magnification (×40). CD34-positive endothelial cells or clusters, which appeared brownish-yellow or brown and were clearly distinguishable from adjacent tumor cells and connective tissue, were counted. The average of three high-power fields was recorded as the microvessel density (MVD) for each sample (20).

### 2.4 Statistical analysis

All statistical analyses were conducted using OriginPro 2025 software. Quantitative data conforming to a normal distribution are presented as mean ± standard deviation, and comparisons between two groups were performed using the Student’s *t*-test. For non-normally distributed data, values are expressed as median (M) and interquartile range (IQR), with comparisons between groups conducted using the Mann–Whitney *U* test. For comparisons among three groups, one-way analysis of variance was applied, followed by least significant difference (LSD) *post hoc* pairwise comparisons to test individual intergroup differences when the overall ANOVA *p*-value was less than 0.05. A *p*-value of less than 0.05 was considered indicative of statistical significance.

## 3 Results

### 3.1 General condition of animals

All procedures were completed successfully without intraoperative complications. The mean operative time was significantly shorter in the Experimental Group compared to the Surgical Group (*p* < 0.05). Postoperatively, normal feeding behavior and activity levels were observed in both groups. No severe infections or other complications occurred, and a 100% survival rate was achieved. Lesion modeling was successful in 18 of 20 nodules in the Experimental Group and in 19 of 20 lesions in the Surgical Group, corresponding to success rates of 90.0 and 95.0%, respectively. The difference in modeling success rates between the two groups was not statistically significant (*p* > 0.05).

### 3.2 Pathological features

Gross examination presented no significant differences in lesion appearance between the two groups. Most nodules were ovoid, although some exhibited irregular contours. The nodules appeared milky-white in color and were slightly firmer than the surrounding hepatic tissue. Cut surfaces were uniform, with clear demarcation from adjacent liver parenchyma, and irregular necrotic foci were noted in some lesions (Figure 2A).

**Figure 2:**
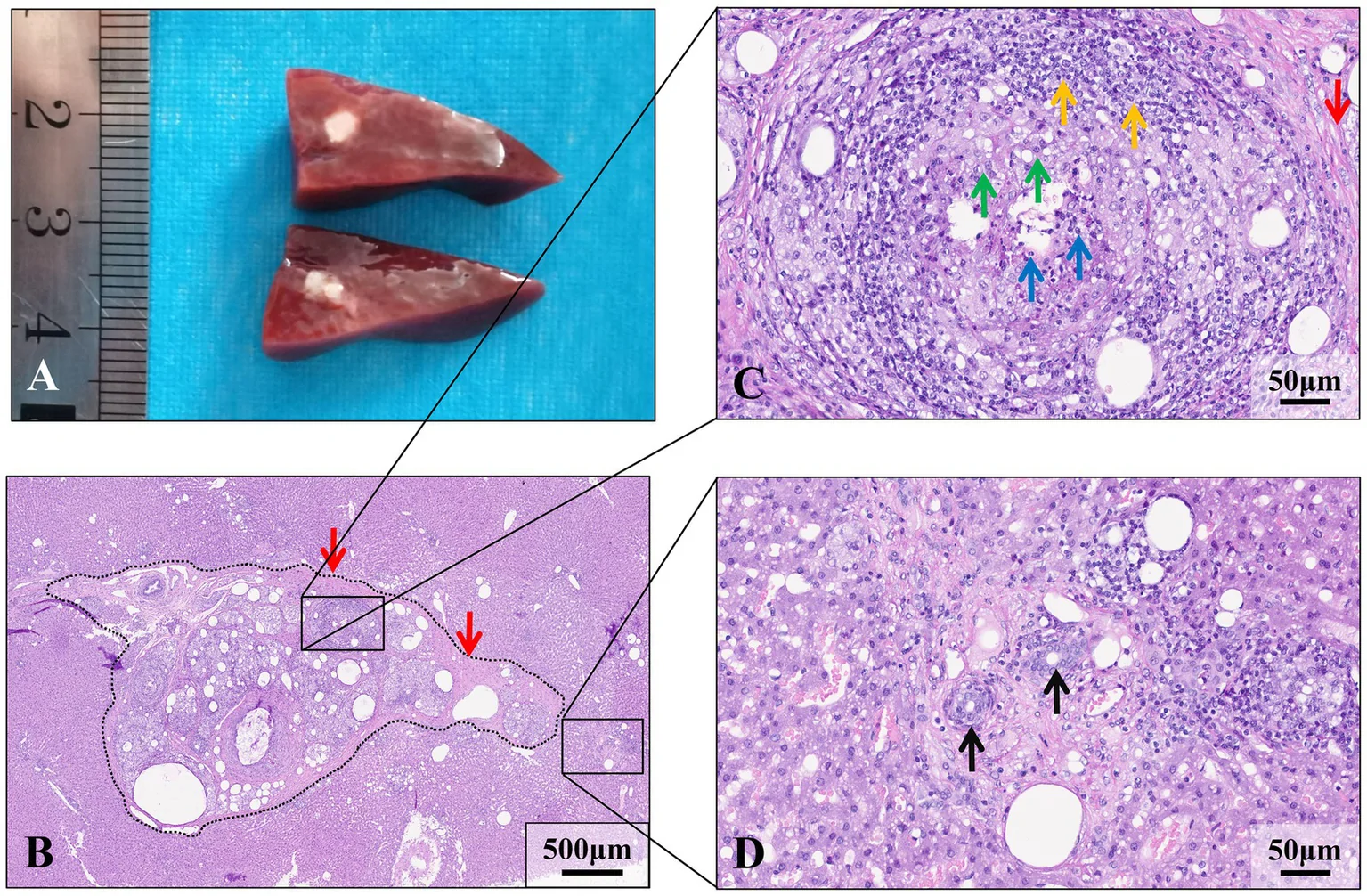
Pathological features of the rabbit IPTL model. **(A)** Gross view showing white nodular IPTL lesions with uniform texture and clear demarcation from the surrounding liver parenchyma. **(B)** H&E staining, ×5: lightly stained inflammatory pseudotumor with multiple intralesional inflammatory granulomas, surrounded by fibrous tissue (red→). **(C)** H&E staining, ×40: intralesional inflammatory granulomas containing histiocytes (green→), neutrophils (blue→), lymphocytes, and plasma cells (orange→). **(D)** Inflammatory cell infiltration around adjacent small bile ducts (black→).

Histological analysis confirmed features consistent with IPTL in both groups. The nodules were composed of proliferative granulation tissue encased by fibrous tissue, with prominent internal fibrosis and infiltration by mixed inflammatory cells, including multinucleated cells. Areas of hepatocellular degeneration or necrosis were identified, along with irregular, structureless necrotic regions. Surrounding hepatic tissue exhibited pronounced inflammatory or fibrotic reactions, with abundant vascular structures observed in the peripheral inflammatory zones. Inflammatory cell infiltration was also noted around small bile ducts adjacent to the lesions (Figures 2B–D).

### 3.3 Conventional ultrasound

On B-mode ultrasonography, lesions in both the Experimental and Surgical Groups appeared as hyperechoic nodules with predominantly well-defined margins, although some demonstrated irregular shapes. CDFI indicated focal or sparse intralesional vascular signals in a subset of nodules. No significant differences were observed between the two groups regarding modeling success rate, lesion diameter, echogenicity, morphology, or intralesional blood flow signals. Statistical analysis confirmed the absence of significant intergroup differences in blood flow patterns (Table 1).

**Table 1:** Features of IPTL lesions in the two groups.

Features	Experimental Group	Surgical Group	t/χ^2^	*P*
Modeling outcomes				
Success rate, *n* (%)	18 (90%)	19 (95%)	-	1.000
Procedure time(s)	21.10 ± 3.67	32.10 ± 4.09	6.330	0.000
Diameter (mm)	9.88 ± 3.90	10.70 ± 3.90	0.637	0.528
MVD	16.06 ± 4.04	17.95 ± 2.53	1.719	0.094
US characteristics				
Blood flow grade				
0	9	6		
1	5	7		
2	2	5		
3	2	1	2.527	0.470
Shape				
Oval/round	10	13		
Irregular	8	6	0.222	0.638
Margin				
Circumscribed	17	17		
Indistinct	1	2		0.405
Enhancement patterns				
Type I- Isoenhancement	10	9		
Type II- Hypoenhancement	4	6		
Type III- Rapid Washout	4	4	0.426	0.808

US = ultrasound.

### 3.4 CEUS findings

CEUS revealed that the majority of lesions demonstrated homogeneous enhancement, although non-enhancing necrotic foci were observed in some lesions. Based on enhancement characteristics, nodules were classified into three types: Type I (*n* = 19), iso-enhancement lesions exhibited contrast enhancement comparable to the adjacent liver parenchyma throughout the arterial, portal, and delayed phases, with indistinct boundaries (Figures 3A1–3); Type II (*n* = 10), hypoenhancing lesions showed reduced enhancement relative to the liver parenchyma across all imaging phases (Figures 3B1–3); and Type III (*n* = 8), rapid washout lesions presented with iso-enhancement in the early arterial phase, followed by early contrast washout during the late arterial phase, and markedly reduced enhancement during the portal and delayed phases compared to the liver parenchyma (Figures 3C1–3).

**Figure 3:**
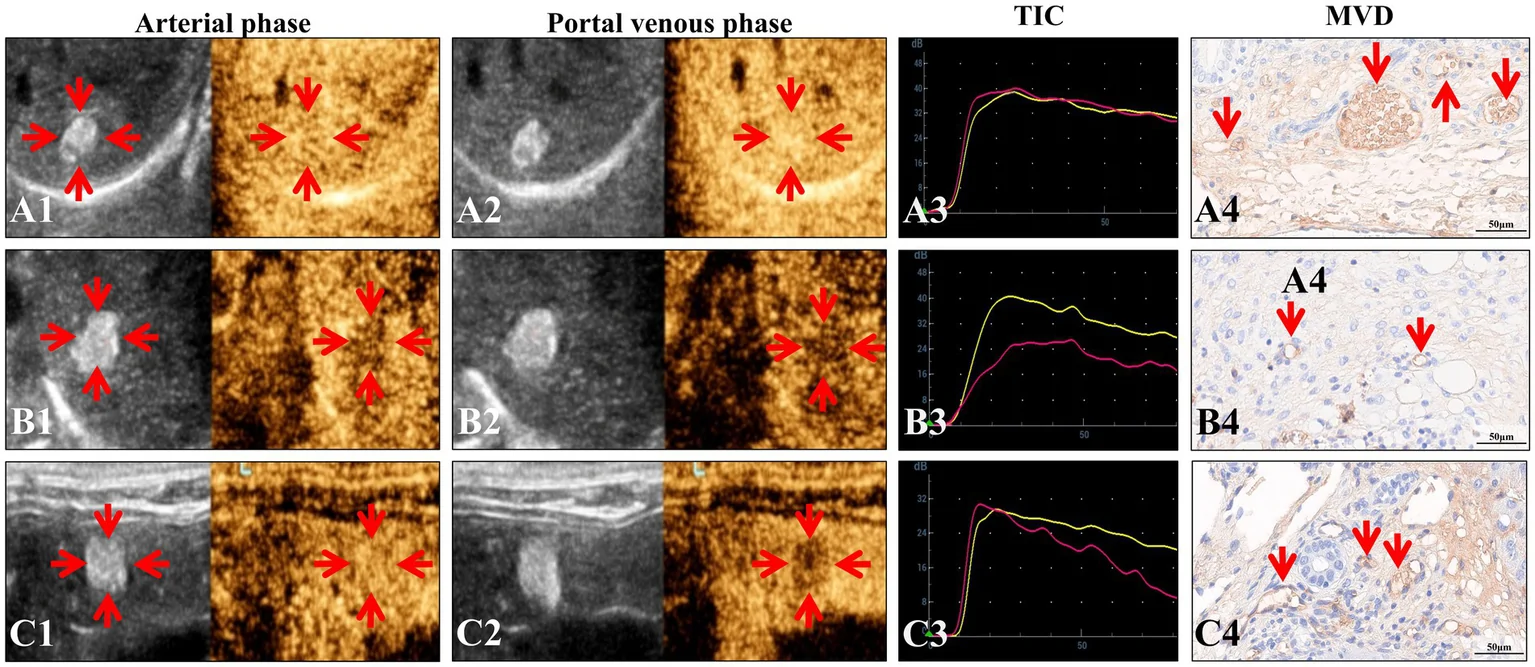
CEUS features of rabbit IPTL with corresponding histopathology **(A1,2)** Lesions show isoenhancement during the arterial and portal phases. **(A3)** TICs demonstrated comparable enhancement kinetics between isoenhancing IPTL lesions and the surrounding liver parenchyma. The horizontal axis represents time(s), and the vertical axis denotes signal intensity (decibel, dB). **(A4)** Numerous microvessels are visible within the lesion (→), with a MVD of 20. **(B1,2)** Lesions exhibit hypoenhancement during the arterial and portal phases. **(B3)** TICs demonstrated persistent hypoenhancement of the lesions compared with the surrounding liver parenchyma. **(B4)** Sparse microvessels are observed within the lesion (→), with an MVD of 8. **(C1,2)** Lesions display isoenhancement in the arterial phase and hypoenhancement in the portal phase. **(C3)** TICs demonstrated early enhancement comparable to the surrounding liver parenchyma, followed by rapid washout. **(C4)** Several microvessels are present within the lesion (→), with an MVD of 17.

Quantitative analysis revealed no significant differences in PI, AT, RT, TTP, or WT between the lesions (in either experimental group) and the surrounding liver parenchyma (all *p* > 0.05) (Table 2). Subgroup analysis based on enhancement patterns further revealed that Type II nodules exhibited significantly lower PI (23.45 ± 4.62 dB) and MVD (14.50 ± 3.14) compared with both Type I (PI: 29.98 ± 6.89 dB; MVD: 18.21 ± 3.22) and Type III (PI: 30.80 ± 6.40 dB; MVD: 17.63 ± 2.92) lesions. For PI, LSD *post hoc* analysis demonstrated that PI was significantly lower in Type II lesions than in Type I (*p* = 0.012) and Type III lesions (*p* = 0.019). Similarly, LSD *post hoc* analysis showed that MVD was significantly lower in Type II lesions compared with Type I (*p* = 0.005) and Type III lesions (*p* = 0.043) (Table 3).

**Table 2:** Comparison of CEUS parameters between IPTL lesions and background liver parenchyma.

Parameters	Experimental group	Surgical group	Liver parenchyma (Blank control)	*F*	*P*
PI (db, $\bar{x}\pm s$ )	28.73 ± 7.26	28.03 ± 6.50	29.40 ± 7.02	0.155	0.857
AT (s, $\bar{x}\pm s$ )	11.92 ± 3.05	11.34 ± 3.04	13.99 ± 4.14	2.634	0.082
RT (s, $\bar{x}\pm s$ )	16.96 ± 5.16	17.62 ± 5.58	22.18 ± 8.49	2.650	0.083
TTP (s, $\bar{x}\pm s$ )	31.17 ± 8.48	32.43 ± 7.36	36.17 ± 10.51	1.371	0.264
WT (s, $\bar{x}\pm s$ )	131.03 ± 23.48	123.85 ± 23.25	119.44 ± 25.09	0.976	0.384

**Table 3:** Comparison of Parameters Stratified by CEUS Enhancement Patterns.

Parameter	Type I-Isoenhancement *n* = 19	Type II-Hypoenhancement *n* = 10	Type III-Rapid washout *n* = 8	*F*	*P*
Lesion characteristics					
MVD	18.21 ± 3.22	14.50 ± 3.14*	17.63 ± 2.92	4.723	0.015
Size(mm)	9.72 ± 3.44	10.95 ± 4.42	11.08 ± 4.18	0.488	0.618
CEUS parameters					
PI(db, $\bar{x}\pm s$ )	29.98 ± 6.89	23.45 ± 4.62*	30.80 ± 6.40	4.312	0.021
AT(s, $\bar{x}\pm s$ )	12.57 ± 3.58	10.84 ± 0.40	10.06 ± 0.70	2.666	0.084
RT(s, $\bar{x}\pm s$ )	13.00 ± 5.47	16.39 ± 5.08	17.91 ± 6.87	2.032	0.151
TTP(s, $\bar{x}\pm s$ )	31.34 ± 8.15	34.82 ± 7.50	30.61 ± 7.22	0.844	0.439
WT(s, $\bar{x}\pm s$ )	121.70 ± 23.38	132.59 ± 21.89	132.78 ± 23.33	1.053	0.360

*, *Post hoc* pairwise comparisons showed a significantly lower mean value compared to the other two groups.

### 3.5 Laboratory tests

Comparative analysis of serum biomarkers including HA, AST, ALT, and TBIL indicated that HA levels in the Surgical Group were significantly elevated compared to those in the blank control group. No significant differences were identified among the groups for the remaining parameters (Figure 4).

**Figure 4:**
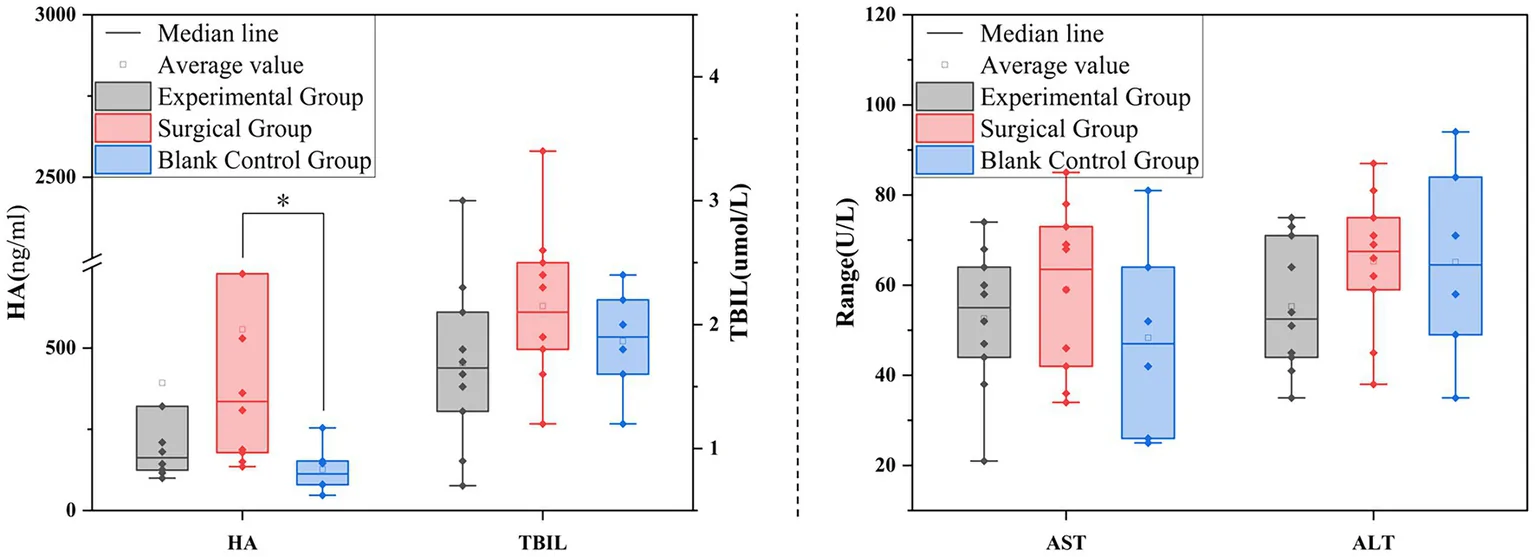
Laboratory test results of the three groups of rabbits. *p < 0.05.

## 4 Discussion

IPTL is an uncommon, benign hepatic lesion with poorly understood etiology, pathogenesis, and imaging features due to the limited number of reported cases and absence of large-scale studies (21). To address this limitation, we developed a modified ultrasound-guided IPTL rabbit model that improves upon previously reported approaches by introducing a minimally invasive implantation strategy and by characterizing CEUS heterogeneity. To our knowledge, this is the first reported ultrasound-guided rabbit IPTL model characterized by heterogeneous CEUS enhancement patterns, thereby enhancing its potential for translational research.

### 4.1 Development and innovation of IPTL animal models

FCA, first formulated by Jules Freund in the 1940s, contains a complex mixture of lipids, proteins, and polysaccharides and is capable of nonspecifically stimulating the host immune response to antigens. However, its administration may lead to local granuloma formation and other inflammatory reactions (22). Early studies attempted to establish rabbit models of IPTL through direct FCA injection into hepatic tissue (23, 24). Although diffuse inflammatory lesions were induced, these attempts did not result in the formation of localized IPTL nodules. Subsequently, surgical techniques involving the implantation of gelatin sponge blocks followed by perilesional FCA injection were adopted (14, 15, 25). These approaches successfully produced rabbit IPTL models, thereby facilitating further investigations like ultrasound assessment and histopathological examination. One study also applied this surgical embedding method to compare imaging features between IPTL and VX2 tumor models, further supporting its utility (13).

Both approaches demonstrated high modeling success rates and favorable biosafety profiles. In recent years, advancements in ultrasound-guided interventional techniques have introduced new possibilities for the construction of IPTL models (26). In the present study, a modified method was implemented involving the ultrasound-guided implantation of gelatin sponge particles into the rabbit liver, followed by localized FCA injection. This method significantly reduced operative time, minimized surgical trauma, and was not associated with severe complications.

Postoperative assessments showed that HA levels were significantly elevated in the Surgical Group compared with the blank control group, whereas no significant changes in HA, AST, ALT, or TBIL were observed in the Experimental Group. As HA is involved in extracellular matrix remodeling and may increase during hepatic tissue repair, the elevated HA level in the Surgical Group may partly reflect additional tissue injury and repair responses caused by surgical manipulation. The absence of HA elevation in the ultrasound-guided group, together with the shorter procedure time and lack of surgical complications, suggests that this approach may reduce procedure-related hepatic injury. However, HA alone cannot fully distinguish trauma-induced changes from specific fibrotic responses, and further studies incorporating additional fibrosis markers and histopathological evaluation are warranted (27, 28). These findings indicate that the ultrasound-guided approach causes less short-term procedure-related hepatic injury and has a favorable short-term safety profile. Instances of modeling failure were likely attributable to postoperative displacement of gelatin sponge particles into the peritoneal cavity, thereby preventing the formation of focal lesions at the intended hepatic site.

### 4.2 Diverse CEUS enhancement patterns and their significance in IPTL

No significant differences were identified between the Experimental and Surgical Groups in terms of conventional ultrasound, CDFI, or CEUS findings. Histopathological evaluation confirmed that lesions in the Experimental Group exhibited typical features of IPTL, comparable to those observed in the Surgical Group. On conventional ultrasound, all IPTL nodules appeared as ovoid or irregular hyperechoic masses, most of which had well-defined margins, although a minority displayed indistinct boundaries. Posterior acoustic enhancement was generally not prominent. Approximately half of the nodules showed no detectable intralesional blood flow on CDFI, while the remaining lesions exhibited varying degrees of vascular signals, consistent with findings reported in prior studies (13–15).

Earlier studies involving rabbit models of IPTL reported homogenous CEUS enhancement patterns that were indistinguishable from adjacent liver parenchyma (13, 14). In contrast, the present study identified multiple CEUS enhancement patterns within IPTL nodules, marking the first reported instance of such diversity in IPTL models. Although non-enhancing IPTL lesions have been previously described (29), the concurrent presence of several enhancement types within a unified experimental framework has not been previously reported.

The variability in CEUS findings may be attributed to differences in lesion maturation time. Earlier studies employed a 2-week development period, whereas a 3-week interval was applied in the current study, potentially allowing for more complete lesion maturation and the development of diverse enhancement characteristics. The isoenhancing lesions observed in this study may indicate relatively preserved perfusion similar to the surrounding liver parenchyma. This observation was consistent with the relatively preserved microvascular structures observed histologically. The heterogeneity observed in CEUS enhancement patterns highlights the potential value of this model for investigating imaging–pathology relationships in IPTL.

### 4.3 Correlation between CEUS enhancement patterns and histopathological features of IPTL

CEUS demonstrated heterogeneous enhancement patterns in IPTL lesions, including isoenhancement, hypoenhancement, and rapid washout patterns. This variability may reflect differences in inflammatory activity, tissue remodeling, and microvascular distribution during IPTL development. IPTL lesions consist of various components, including inflammatory cell infiltration, fibrosis, and variable angiogenesis, which may result in heterogeneous perfusion characteristics (30). Consistent with methodological observations in preclinical liver imaging models, contrast-enhanced ultrasound provides a reliable *in vivo* approach to characterize such microvascular perfusion heterogeneity at the functional level (31).

In our study, hypoenhancement lesions showed significantly lower PI values and reduced MVD compared with other enhancement patterns, suggesting that insufficient microvascular perfusion may contribute to decreased enhancement intensity. In contrast, lesions with isoenhancement or rapid washout patterns exhibited relatively higher microvascular density, which may be associated with preserved or enhanced local blood supply caused by inflammatory vascular remodeling. Previous studies have indicated that inflammatory processes and tissue repair responses can induce microvascular changes and extracellular matrix remodeling, potentially influencing CEUS manifestations (32).

The presence of multiple enhancement patterns highlights the complexity of IPTL microcirculation and suggests that CEUS may provide valuable information regarding intralesional vascular characteristics and pathological heterogeneity.

### 4.4 Limitations

Several limitations of this study should be acknowledged. First, the two-dimensional ultrasonographic appearance of IPTL in clinical practice is heterogeneous, with lesions presenting as either hyperechoic or hypoechoic nodules. In contrast, all lesions in the present model were hyperechoic, which may not fully represent the variability observed in human IPTL. Second, although clinical studies have described multiple pathological subtypes of IPTL, the induced lesions in this model mainly correspond to the proliferative subtype characterized by limited arterial blood supply. Third, IPTL in humans generally has a favorable prognosis, with spontaneous regression or resolution after treatment; however, the long-term outcomes and natural course of the modeled lesions were not evaluated, leaving their temporal behavior undefined. Fourth, the potential correlation between the two lesions from the same animal was not explicitly incorporated into lesion-level statistical comparisons, which may slightly lessen the statistical stringency of lesion-level results. Finally, although this study demonstrated model feasibility and correlations between CEUS patterns and microvascular features, the inflammatory microenvironment—including inflammatory cell subpopulations, fibrotic remodeling, and molecular biomarkers—was not comprehensively assessed and warrants further investigation.

## 5 Conclusion

In summary, both the ultrasound-guided implantation and surgical embedding methods successfully produced IPTL lesions exhibiting multiple CEUS enhancement patterns, showing diverse CEUS enhancement patterns relevant to IPTL imaging evaluation. The ultrasound-guided implantation method, however, was associated with low surgical trauma, minimal hepatic injury, decreased procedural duration while maintaining comparable modeling reproducibility. These attributes support the use of this approach as a reliable and efficient platform for experimental IPTL modeling. The ability to reproduce varied CEUS enhancement patterns enhances the translational value of the model, offering a robust foundation for future studies on the imaging–pathology correlations and differential diagnosis of IPTL.

## Data Availability

The raw data supporting the conclusions of this article will be made available by the authors, without undue reservation.

## References

[1] Wang D Misdraji J. Inflammatory pseudotumor of the liver. Surg Pathol Clin. (2023) 16:565–80. doi: 10.1016/j.path.2023.04.010 37536889

[2] Sedlic T Scali EP Lee WK Verma S Chang SD. Inflammatory pseudotumours in the abdomen and pelvis: a pictorial essay. Can Assoc Radiol J. (2014) 65:52–9. doi: 10.1016/j.carj.2013.02.003, 23830343

[3] Chang SD Scali EP Abrahams Z Tha S Yoshida EM. Inflammatory pseudotumor of the liver: a rare case of recurrence following surgical resection. J Radiol Case Rep. (2014) 8:23–30. doi: 10.3941/jrcr.v8i3.1459, 24967025 PMC4035365

[4] Calistri L Maraghelli D Nardi C Vidali S Rastrelli V Crocetti L . Magnetic resonance imaging of inflammatory pseudotumor of the liver: a 2021 systematic literature update and series presentation. Abdom Radiol (NY). (2022) 47:2795–810. doi: 10.1007/s00261-022-03555-9, 35648207 PMC9300573

[5] Gesualdo A Tamburrano R Gentile A Giannini A Palasciano G Palmieri V. A diagnosis of inflammatory pseudotumor of the liver by contrast Enhaced ultrasound and fine-needle biopsy: a case report. Eur J Case Rep Intern Med. (2017) 4:000495. doi: 10.12890/2016_000495, 30755918 PMC6346893

[6] Barabino M Piccolo G Tramacere A Volponi S Cigala C Gianelli U . Inflammatory pseudotumor of the liver or intrahepatic cholangiocarcinoma, that's the question: a review of the literature. Cancers (Basel). (2024) 16:2926. doi: 10.3390/cancers16172926, 39272784 PMC11394391

[7] Li M Sobnach S Kotze UK Zerbini LF Millis JM Hampton DA. Inflammatory myofibroblastic tumours of the liver-a systematic review. S Afr J Surg. (2024) 62:23–7. 38838115

[8] Oh K Hwang S Ahn CS Kim KH Moon DB Ha TY . Clinicopathological features and post-resection outcomes of inflammatory pseudotumor of the liver. Ann Hepatobiliary Pancreat Surg. (2021) 25:34–8. doi: 10.14701/ahbps.2021.25.1.34, 33649252 PMC7952659

[9] Chinese Society of Ultrasound in Medicine, Oncology Intervention Committee of Chinese Research Hospital Society, National Health Commission Capacity Building and Continuing Education Expert Committee on Ultrasonic Diagnosis. Guideline for ultrasonic diagnosis of liver diseases. Chin J Hepatol. (2021) 29:385–402. doi: 10.3760/cma.j.cn501113-20210219-00087 PMC12813929 34107574

[10] Barr RG. Contrast enhanced ultrasound for focal liver lesions: how accurate is it? Abdom Radiol (NY). (2018) 43:1128–33. doi: 10.1007/s00261-017-1257-6, 28718042

[11] Kong WT Wang WP Shen HY Xue HY Liu CR Huang DQ . Hepatic inflammatory pseudotumor mimicking malignancy: the value of differential diagnosis on contrast enhanced ultrasound. Med Ultrason. (2021) 23:15–21. doi: 10.11152/mu-2542, 32905565

[12] Samanta A Sen Sarma M. Hepatic pseudotumor: a diagnostic challenge. World J Hepatol. (2024) 16:667–70. doi: 10.4254/wjh.v16.i5-.667 PMC11135271 38818301

[13] Shi Y Wang X Qiao Y Bai X Wang Q Lei C. The dynamic enhanced characterization with low mechanical index gray-scale harmonic imaging inflammatory pseudotumor of liver compared with hepatic VX2 tumor and normal liver. Adv Clin Exp Med. (2020) 29:1073–81. doi: 10.17219/acem/110315 32886457

[14] Wang XH Bai XP Shi Y Wang QX Qiao YY Lei CG. Hepatic inflammatory pseudotumor: hemodynamic changes assessed by contrast-enhanced ultrasound timeintensity curves in an experimental rabbit study. Chin J Med Ultrasound (Electronic Version). (2007) 2:72–4.

[15] Wang QX Lei CG Wang XH Bai XP Shi Y Qiao YY. Comparative study of real-time enhanced ultrasonography and pathology in the diagnosis of hepatic inflammatory pseudotumor in rabbits. Chin J Ultrasound Med. (2015) 31:71–3.

[16] Liu ZL Lu Q Zhang Q Fan WW Chen J Liu LP. NAFLD accelerates arterial perfusion in rabbit VX2 liver tumors: a sonoVue-based contrast-enhanced ultrasound study. Front Oncol. (2025) 15:1638869. doi: 10.3389/fonc.2025.1638869, 40994954 PMC12454042

[17] Zhang YJ Liu LP Lu Q . Establishment of inflammatory pseudotumor of liver modified model in rabbits with fatty liver and normal liver showed on ultrasound imaging. Natl Med Front China. (2013) 8:27–8. doi: 10.3969/j.issn.1673-5552.2013.05.0012

[18] Adler DD Carson PL Rubin JM Quinn-Reid D. Doppler ultrasound color flow imaging in the study of breast cancer: preliminary findings. Ultrasound Med Biol. (1990) 16:553–9. doi: 10.1016/0301-5629(90)90020-d, 2238263

[19] Yang HK Burns PN Jang HJ Kono Y Khalili K Wilson SR . Contrast-enhanced ultrasound approach to the diagnosis of focal liver lesions: the importance of washout. Ultrasonography. (2019) 38:289–301. doi: 10.14366/usg.19006, 31311068 PMC6769186

[20] Pandey S Shukla S Vagha S. Utility of CD34 in assessing microvessel density and its correlation with clinicopathological parameters in colorectal carcinoma patients. Cureus. (2023) 15:e49186. doi: 10.7759/cureus.49186, 38130549 PMC10734711

[21] Ichikawa S Motosugi U Suzuki T Shimizu T Onishi H. Imaging features of hepatic inflammatory pseudotumor: distinction from colorectal liver metastasis using gadoxetate disodium-enhanced magnetic resonance imaging. Abdom Radiol (NY). (2020) 45:2400–8. doi: 10.1007/s00261-020-02575-7, 32468212

[22] Hirschowitz D Fox A Glynn LE. Effect of excising Freund's adjuvant granuloma on subsequent development of experimental allergic arthritis. Ann Rheum Dis. (1977) 36:381–2. doi: 10.1136/ard.36.4.381, 901038 PMC1006703

[23] Shi Y Bai XP Wang XH Wang QX Qiao YY Lei CG . Establishment of hepatic inflammatory pseudotumor modified model in rabbits showed on ultrasound imaging. Chin J Ultrasound Med. (2007) 10:724–6.

[24] Li J Dong B Yu X Li C. Ultrasonographic portography with low mechanical index gray-scale imaging in hepatic VX2 tumor. Ultrasound Med Biol. (2006) 32:641–7. doi: 10.1016/j.ultrasmedbio.2006.01.008 16677923

[25] Liu LP Li YP Lu Q . Comparative study on blood perfusion and angiogenesis of intrahepatic VX2 tumors and inflammatory pseudotumor by contrast-enhanced ultrasound in rabbits with fatty liver. Chin J HepatlibiarySurg. (2016) 22:766–70. doi: 10.3760/cma.j.issn.1007-8118.2016.11.014

[26] Nielsen MB Søgaard SB Bech Andersen S Skjoldbye B Hansen KL Rafaelsen S . Highlights of the development in ultrasound during the last 70 years: a historical review. Acta Radiol. (2021) 62:1499–514. doi: 10.1177/02841851211050859, 34791887

[27] Kim J Seki E. Hyaluronan in liver fibrosis: basic mechanisms, clinical implications, and therapeutic targets. Hepatol Commun. (2023) 7:e 0083. doi: 10.1097/HC9.0000000000000083, 36930869 PMC10027054

[28] Tamber SS Bansal P Sharma S Singh RB Sharma R. Biomarkers of liver diseases. Mol Biol Rep. (2023) 50:7815–23. doi: 10.1007/s11033-023-08666-0, 37482588

[29] Zhang YJ Liu LP Lu Q . Comparative study of inflammatory pseudoneoplasm of the liver in rabbits with fatty liver and normalliver by contrast-enhanced ultrasonography. Chin J Ultrasonogr. (2013) 22:525–9. doi: 10.3760/cma.j.issn.1004-4477.2013.06.022

[30] Tan Z Sun H Xue T Gan C Liu H Xie Y . Liver fibrosis: therapeutic targets and advances in drug therapy. Front Cell Dev Biol. (2021) 9:730176. doi: 10.3389/fcell.2021.730176, 34621747 PMC8490799

[31] Ghosh D Kumar A. Ultrasound imaging in orthotopic hepatocellular carcinoma models: promise, practicality, and points for refinement. World J Hepatol. (2025) 17:115551. doi: 10.4254/wjh.v17.i12.115551, 41479514 PMC12754439

[32] Wu L Wu Y Jin J . An optimal prognostic model based on multiparameter ultrasound for acute-on-chronic liver failure. Ultrasound Med Biol. (2023) 49:2183–90. doi: 10.1016/j.ultrasmedbio 37451951

